# Developmental outcomes of very low birth weight infants with catch-up head growth: a nationwide cohort study

**DOI:** 10.1186/s12887-023-04135-6

**Published:** 2023-08-08

**Authors:** You Mi Hong, Dong Hue Cho, Jin Kyu Kim

**Affiliations:** 1 Department of Obstetrics and Gynecology, University of Ulsan College of Medicine, Asan Medical Center, Seoul, Korea; 2 Research Institute of Clinical Medicine of Jeonbuk National University–Biomedical Research Institute of Jeonbuk National University Hospital, Jeonju, Korea; 3 Department of Obstetrics and Gynecology, Jeonbuk National University School of Medicine, Jeonju, Korea; 4 Department of Pediatrics, Jeonbuk National University School of Medicine, Jeonju, Korea

**Keywords:** Bronchopulmonary dysplasia, Catch-up head growth, Developmental delay, Head circumference, Very low birth weight

## Abstract

**Background:**

As the survival rates of very low birth weight (VLBW) infants have increased, their neurodevelopmental outcomes are of concern. This study aims to determine the demographic and perinatal characteristics of premature infant according to head growth, identify clinical factors affecting growth catch-up, and explore differences in developmental outcomes according to catch-up states.

**Methods:**

This nationwide prospective cohort study of Korean Neonatal Network data analyzed premature infants with very low birth weight (< 1,500 g) between 2014 and 2017. A total of 253 eligible infants who had completed the Bayley Scales of Infant and Toddler Development, Third Edition, were assigned into two groups: a catch-up (CU) group with a head circumference above the 10^th^ percentile and a no catch-up (NCU) group with a head circumference below the 10^th^ percentile at 18–24 months of corrected age (CA).

**Results:**

Most (81.4%, 206/253) premature infants exhibited catch-up growth at 18–24 months of CA. Rates of microcephaly, intraventricular hemorrhage (IVH), bronchopulmonary dysplasia (BPD), sepsis, necrotizing enterocolitis (NEC), length of NICU stay, ventilation care, and parenteral nutrition were significantly greater in the NCU group (*P* < 0.05). On multiple linear regression analysis, BPD status was the most influential clinical factor affecting catch-up head growth after adjusting for gestational age, birth weight, and birth head circumference (adjusted OR 4.586, 95% CI 1.960–10.729). At 18–24 months of CA, the NCU group exhibited lower developmental indices and a higher rate of developmental delay than the CU group. Motor developmental delay was the most significant factor relevant to catch-up head growth, and the motor development difference between the two groups was only statistically significant after adjusting for four major neonatal morbidities: IVH, BPD, sepsis, and NEC status (adjusted OR 10.727, 95% CI 1.922–59.868).

**Conclusion:**

As association was observed between head growth catch-up status and developmental outcomes in VLBW infants at 18–24 months of CA. Key clinical factors associated with catch-up status included BPD and NEC status, length of parenteral nutrition, and ventilator care. Further study is needed to establish causality and explore additional factors that may influence developmental outcomes in this population.

## Introduction

Recent advances in neonatal and neonatal intensive care have improved infant survival rates. In Korea, the survival rate of very low birth weight (VLBW) infants has also increased from 34% in the 1960s to 77.5% in the 2000s [1]. However, VLBW remains a major cause of infant mortality and morbidity. For this reason, there are concerns that the improved survival of infants with VLBW might be accompanied by an increased of disabling morbidity in survivors. While the incidence of severe cerebral palsy, blindness, and hearing impairment have decreased over time, cognitive impairments have become more prevalent sequelae in VLBW children [2–5]. Although premature birth in itself might adversely affect later development, insight into factors influencing cognitive outcomes is key to improving such outcomes. However, few studies have reported the correlation between developmental outcome and head growth in VLBW infants. The Korean Neonatal Network (KNN) is a nationwide, multicenter, prospective, web-based cohort registry system for VLBW infants with a birth weight less than 1,500 g. This study aims to determine the demographic and perinatal characteristics of premature infant according to head growth, identify clinical factors affecting growth catch-up, and find differences in developmental outcomes according to catch-up states based on the KNN cohort data.

## Methods

### Patients

**Fig. 1:**
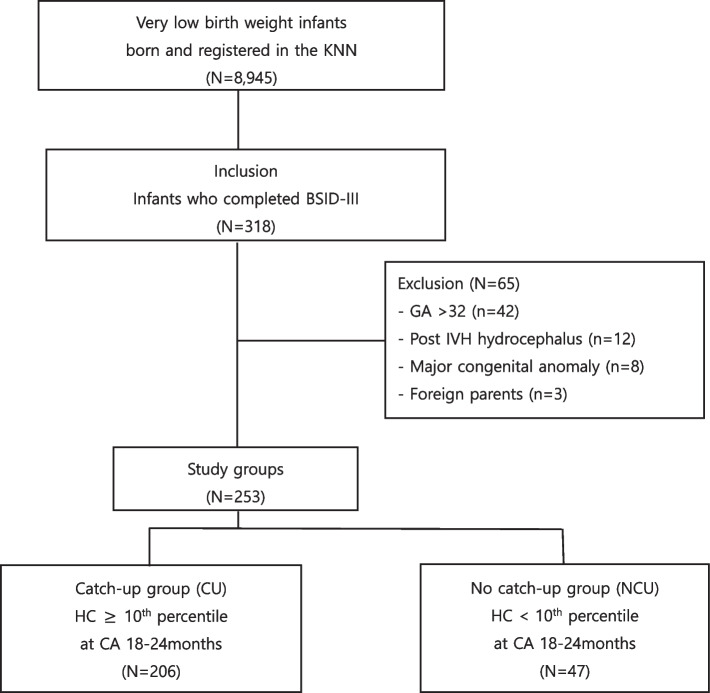
Flowchart outlining the study population selection. This study enrolled 318 VLBW infants. After excluding 65 infants, the remaining infants (*n* = 253) who had completed the Bayley Scales of Infant and Toddler Development (BSID), Third Edition, were assigned into two groups: a catch-up (CU) group with a head circumference above the 10^th^ percentile and a no catch-up (NCU) group with a head circumference below the 10^th^ percentile at 18–24 months of corrected age (CA). Abbreviations: KNN, Korean Neonatal Network; BSID, Bayley Scales of Infant and Toddler Development; GA, Gestational age; IVH, Intraventricular hemorrhage; HC, Head circumference; CA. Corrected age

Of 8,945 VLBW infants born between January 2014 and November 2017 and registered in the database registry of KNN, 318 with completed Bayley scales of infant and toddler development (BSID) III at 18–24 months of corrected age (CA) were selected for this study. The BSID test was revised and reconstructed into the third edition, which included a separation of the mental developmental index into language scale. A total 65 infants were excluded due to gestational age (GA) > 32 weeks (42 infants), the presence of major congenital anomalies (8 infants), non-Korean (3 infants) parents, and the presence of post intraventricular hemorrhage (IVH) hydrocephalus (12 infants) (Fig. 1).

Three growth parameters (head circumference, body weight and length) were analyzed for a specific period of time, including at birth, at discharge from the neonatal intensive care unit (NICU), and at 18–24 months of CA. Patients were divided into two groups according to their head circumferences. The first group was the catch-up (CU) group whose head circumference was above the 10^th^ percentile at each measurement period. The second group was the no catch-up (NCU) group whose head circumference was below the 10^th^percentile at 18–24 month of CA. A cutoff was used because previous researches showed the impact of head growth degree and velocity on neurodevelopmental outcomes [6–9].

Four major factors were compared: 1) demographic factors (GA, body weight, small for gestational age [SGA], sex, 1-min and 5-min Apgar scores, and microcephaly); 2) maternal factors (maternal age, cesarean section, and multiple pregnancy, maternal gestational diabetes mellitus [GDM] or overt diabetes mellitus [DM], pregnancy-induced hypertension [PIH] or chronic hypertension [HTN], and premature rupture of membrane [PROM]); 3) neonatal morbidity related factors (neonatal resuscitation, length of NICU stay, ventilator care, oxygen therapy, parenteral nutrition, respiratory distress syndrome [RDS]-status, IVH, bronchopulmonary dysplasia [BPD], periventricular leukomalacia [PVL], patent ductus arteriosus [PDA], neonatal sepsis, necrotizing enterocolitis [NEC], and retinopathy of prematurity [ROP]); and 4) environmental factors (non-parental caregiver, low maternal education, nursery use, and language therapy. All maternal and neonatal variables were compared between the CU and NCU groups at 18–24 months of CA.

### Definitions

A KNN database operation manual was compiled to define patient characteristics. In the manual, GA was determined from the obstetric history based on the last menstrual period. PROM was defined as the rupture of membranes over 24 h before the onset of labor.

RDS was defined as respiratory distress requiring ventilator care with diagnosis based on chest radiographic findings. IVH was defined as grade ≥ 3 according to the classification of Papile et al. Post IVH hydrocephalus was defined as IVH-induced hydrocephalus that required spinal tapping, external ventricular drainage, and/or a shunt operation, except for medical treatment. [10] BPD was defined as the use of more than supplemental oxygen at a 36 weeks’ gestational age, corresponding to moderate to severe BPD using the severity-based definition for BPD of the National Institutes of Health consensus [11]. PVL was defined as cystic PVL based on either head ultrasound or cranial magnetic resonance imaging scans performed at $\ge$2 weeks of age. Symptomatic PDA was defined as clinical symptoms of PDA, such as ventilator dependence, deteriorating respiratory status, increasing recurrent apnea, pulmonary hemorrhage, and hypotension. Early sepsis was defined as a positive blood culture at less than 7 days from birth in symptomatic infants suggestive of septicemia with more than 5 days of antibiotic treatment [12]. NEC was defined as ≥ stage 2b according to the modified Bell criteria [13]. ROP was defined as any ROP that needed anti-vascular endothelial growth factor and/or laser ablative and/or surgical treatment to prevent visual loss [14].

Neonatal resuscitation was defined as the need for initial treatment including oxygen supplementation, positive pressure ventilation, endotracheal intubation, chest compression, and any related medication. The duration of ventilator care was defined as an endotracheal respiratory support by conventional or high-frequency oscillation ventilation. Oxygen therapy was defined as supplemental support with oxygen via a hood, mask, or low-flow nasal cannula. Low maternal education was defined by high school graduation. A language Composite score < 70, or Cognitive Composite score < 70, or Motor Composite score < 70, was defined as a developmental delay on BSID-III [15].

### Statistical analysis

Demographic and perinatal characteristics, head circumferences, and language test results were subjected to frequency analysis. Data are described as median (maximum-minimum) for continuous variables and as numbers for binary and categorical variables. The independent t test and chi square test were used to compare demographic and perinatal characteristics, language developmental results, and clinical characteristics between the CU and NCU groups (*P* < 0.05). Multiple linear regression was employed to determine factors affecting the status of catch-up in head growth. Factors showing statistically significant difference between the two groups were selected and entered into a logistic regression model. When a correlation between clinical factor and catch-up status was found, the adjusted odds ratio was used to offset the impact of extremely early preterm, SGA, and microcephaly instead of the crude odds ratio. These correlations are expressed as an odds ratio with a 95% confidence interval (CI), with a value greater than one indicating increased odds of not achieving catch-up after adjusting for GA, birth weight, and birth head circumference. When comparing developmental outcomes between the two groups, the adjusted odds ratio was used after adjusting for four major neonatal morbidities: IVH, BPD, sepsis, and NEC status. A *P*-value < 0.05 indicated statistical significance. All statistical analyses were performed using SPSS software ver. 26.0 (IBM Corp., Chicago, IL, USA).

## Results

### The distribution of three growth parameters in VLBW infants of 18–24 months of corrected age (CA)

**Fig. 2:**
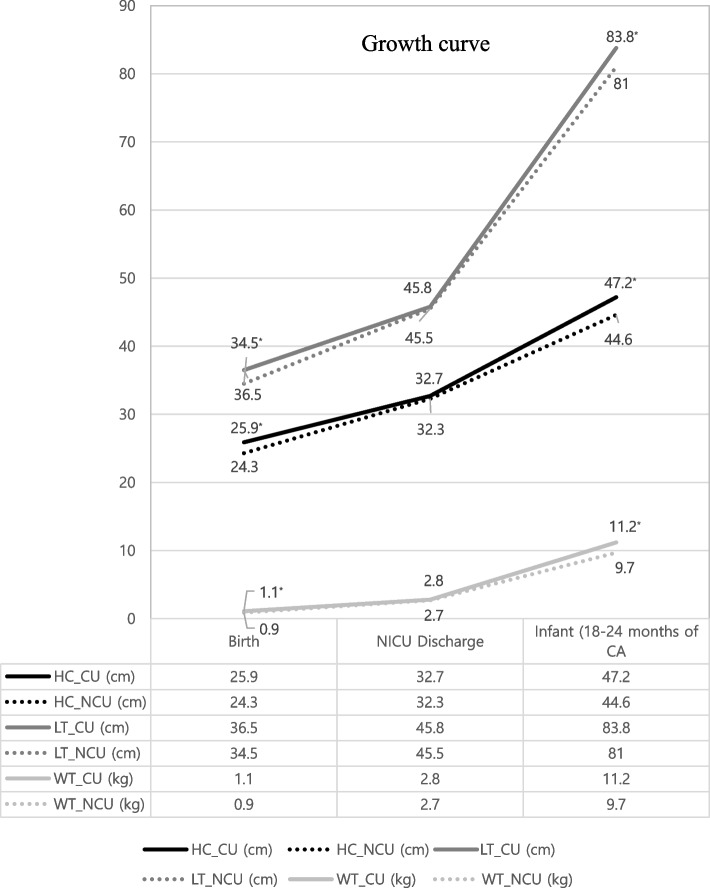
Growth curves of three parameters in premature infants. Infants in the no catch-up (NCU) group had smaller head circumferences, shorter lengths, and lower body weights than those in the catch-up (CU) group. These differences were all statistically significant (*P *< 0.001). Among the three parameters (head circumference, body weight, and length growth), head circumference showed the biggest difference between the two groups, decreasing from birth to discharge. However, it increased from discharge to 18–24 months of CA. Abbreviations: NICU, Neonatal intensive care unit; HC, Head circumference(cm); CU, Catch-up group; NCU, No catch-up group; LT, Length; WT, Weight(kg)

The head circumference, body weight, and length growth states at for VLBW infants at 18–24 months of CA are shown in Table 1. Most (81.4%, 206/253) VLBW infants caught up their head growths at 18–24 months of CA. Infants in the NCU group had smaller head circumferences, shorter lengths, and less body weights than those in the CU group (head circumference: 44.6 cm vs. 47.2 cm; length: 81.0 cm. vs. 83.8 cm; body weight: 9.7 kg vs. 11.2 kg, respectively). These differences were all statistically significant (*P* < 0.001). Among the three parameters, head circumference showed the biggest difference between the two groups, decreasing from birth to discharge. However, it increased from discharge to 18–24 months of CA (1.6 cm, 0.4 cm, and 2.6 cm at birth, discharge, and 18–24 months of CA, respectively) (Fig. 2).

**Table 1:** The distribution of three growth parameters and catch-up status in VLBW infants of 18–24 months of CA

	CU		NCU		Total	*P* value
	N(%)	M ± SD	N(%)	M ± SD	M ± SD (cm)	
HC (cm)	206 (81.4)	47.2 ± 3.5	47 (18.6)	44.6 ± 1.0	46.7 ± 3.4	< 0.001*
LT (cm)		83.8 ± 4.1		81.0 ± 3.5	83.3 ± 4.2	< 0.001*
WT (kg)		11.2 ± 1.4		9.7 ± 1.0	10.9 ± 1.4	< 0.001*

By independent t-test

*Abbreviations*: *CA* Corrected age, *CU* Catch-up group, *NCU* No catch-up group, *M* ± *SD* Mean ± Standard deviations, *HC* Head circumference, *LT* Length, *WT* Weight

^*^P < 0.05 compared with CU

### Comparison of demographic and perinatal characteristics between CU and NCU groups of VLBW infants

The demographic and perinatal characteristics of VLBW infants according to catch-up status are shown in Table 2. Rates of microcephaly, IVH, BPD, NEC, cerebral palsy, length of NICU stay, ventilation care, and parenteral nutrition were significantly greater in the NCU group (microcephaly: 27.7% (13/47) vs. 14.1% (29/206),* P* = 0.015; IVH: 53.2% (25/47) vs. 31.6% (65/206),* P* = 0.005; BPD: 68.1% (32/47) vs. 30.6% (63/206),* P* < 0.001; sepsis: 31.9% (15/47) vs. 17.0% (35/206),* P* = 0.021; NEC: 17.0% (8/47) vs. 3.9% (8/206), *P* < 0.001; NICU stay: 90.6 vs. 74.3, *P* = 0.002; ventilator care: 27.4 vs. 11.9, *P* < 0.001; parenteral nutrition: 40.6 vs. 25.6, *P* < 0.001). Birth weight (BW), rate of low maternal education, and rate of nursery use were significantly lower in the NCU group (BW: 929.9 g vs. 1084.9 g,* P* < 0.001; low maternal education: 6.4% (3/47) vs. 19.4% (40/206), *P* = 0.032; nursery use: 21.3% (10/47) vs. 37.9% (78/206),* P* = 0.031).

**Table 2:** Comparison of demographic and perinatal characteristics between the CU and NCU groups of VLBW infants

	CU (*N* = 206)	NCU (*N* = 47)	*P* value
GA (wk)	27.9 ± 2.0	27.7 ± 2.1	0.466
BW (g)	1084.9 ± 239.1	929.9 ± 264.4	< 0.001*
SGA, n (%)	60 (29.1)	20 (42.6)	0.075
Male sex, n (%)	95 (46.1)	28 (59.6)	0.097
Multiple pregnancy, n(%)	72 (35.0)	16 (34.0)	0.906
Maternal age (y)	33.0 ± 4.0	34.2 ± 3.8	0.052
Cesarean section, n(%)	131 (63.6)	36 (76.6)	0.090
GDM/overt DM, n (%)	13 (6.3)	6 (12.8)	0.131
PIH/chronic HTN, n (%)	46 (22.3)	10 (21.3)	0.876
PROM, n (%)	91 (44.2)	19 (40.4)	0.641
Antenatal steroid use, n (%)	107 (51.9)	24 (51.0)	0.782
1-min Apgar score	4.3 ± 2.0	3.9 ± 1.9	0.223
5-min Apgar score	6.6 ± 1.9	6.4 ± 1.5	0.438
Microcephaly, n (%)	29 (14.1)	13 (27.7)	0.014*
Neonatal resuscitation, n (%)	185 (89.8)	43 (91.5)	0.728
Length of stay in NICU (days)	74.3 ± 32.1	90.6 ± 39.4	0.003*
Ventilator care (days)	11.9 ± 21.2	27.4 ± 26.0	< 0.001*
Oxygen therapy (days)	8.8 ± 10.9	12.9 ± 14.9	0.032*
Postnatal steroid use, n (%)	31 (15.0)	16 (34.0)	0.033*
Parenteral nutrition(days)	25.6 ± 19.5	40.6 ± 25.5	< 0.001*
RDS, n (%)	164 (79.6)	38 (80.9)	0.849
IVH, n (%)	65 (31.6)	25 (53.2)	0.005*
BPD, n (%)	63 (30.6)	32 (68.1)	< 0.001*
PVL, n (%)	12 (5.8)	6 (12.8)	0.096
PDA, n (%)	72 (35.0)	22 (46.8)	0.130
Sepsis, n (%)	35 (17.0)	15 (31.9)	0.020*
NEC, n (%)	8 (3.9)	8 (17.0)	< 0.001*
ROP, n (%)	22 (10.7)	6( 12.8)	0.682
Non parental caregiver, n (%)	7 (3.4)	2 (4.3)	0.776
Low maternal education, n (%)	40 (19.4)	3 (6.4)	0.032*
Nursery use, n(%)	78 (37.9)	10 (21.3)	0.031*
Language therapy, n (%)	6 (2.9)	4 (8.5)	0.076
Rehabilitation therapy, n (%)	61 (29.6)	29 (61.7)	< 0.001*

By independent t-test, chi square test

*Abbreviations*: *CU* Catch-up group, No catch-up group, *VLBW* Very low birth weight, *GA* Gestational age, *BW* Birth weight, *SGA* Small for gestational age, *GDM* Gestational diabetes mellitus, *DM* Diabetes mellitus, *PIH* Pregnancy induced hypertension, *HTN* Hypertension, *PROM* Premature rupture of membranes, *NICU* Neonatal Intensive care unit, *RDS* Respiratory distress syndrome, *IVH* Intraventricular hemorrhage, *BPD* Bronchopulmonary dysplasia, *PVL* Periventricular leukomalacia, *PDA* Patent ductus arteriosus, *NEC* Necrotizing Enterocolitis, *ROP* Retinopathy of Prematurity

^*^P < 0.05 compared with CU

### Correlations between clinical factors and catch-up status of VLBW infants at 18–24 months of CA

After adjusting for three factors (GA, birth weight, and birth head circumference), only IVH, BPD, sepsis, NEC status, length of ventilator care, and parenteral nutrition were significantly associated with catch-up status. BPD status was the most significant clinical factor affecting catch-up head growth (adjusted OR 4.586, 95% CI 1.960–10.729*)* (Table 3).

**Table 3:** Correlations between clinical factors and catch-up status of VLBW infants at 18–24 months of CA

	OR (95% CI)	*P* value	aOR^a^ (95% CI)	*P* value
Length of stay in NICU (days)	1.012 (1.003–1.022)	0.008*	1.011 (0.999–1.024)	0.070
Ventilator care (days)	1.025 (1.012–1.038)	< 0.001*	1.028 (1.010–1.046)	0.002*
Oxygen therapy (days)	1.026 (1.002–1.052)	0.037*	1.012 (0.984–1.041)	0.398
Parenteral nutrition(days)	1.029 (1.015–1.044)	< 0.001*	1.031 (1.013–1.048)	< 0.001*
IVH, n (%)	2.465 (1.295–4.693)	0.006*	2.403 (1.138–5..075)	0.021*
BPD, n (%)	4.842 (2.450–9.569)	< 0.001*	4.586 (1.960–10.729)	< 0.001*
Sepsis, n (%)	2.290 (1.123–4.672)	0.023*	2.380 (1.054–5.371)	0.037*
NEC, n (%)	5.077 (1.797–14.341)	0.002*	4.187 (1.207–14.522)	0.024*

*Abbreviations*: *VLBW* Very low birth weight, *CA* Corrected age, *OR* Odds ratio, *aOR* adjusted odds ratio, *NICU* Neonatal Intensive care unit, *IVH* Intraventricular hemorrhage, *BPD* Bronchopulmonary dysplasia, *NEC* Necrotizing Enterocolitis

By multiple logistic regression analysis

^*^P < 0.05

^a^Adjusted for Gestational age, Birth weight, and Birth head circumference

### Comparison of developmental outcomes between CU and NCU groups of VLBW infants at 18–24 months of CA

At 18**–**24 months of CA, the NCU group exhibited lower developmental indices and higher rate of developmental delay than the CU group (language developmental index: 92.7 ± 14.5 vs. 96.9 ± 15.3, *P* = 0.090, cognitive developmental index: 91.8 ± 14.8 vs. 100.5 ± 14.3, *P* < 0.001*,* motor developmental index: 89.7 ± 17.5 vs. 99.1 ± 12.8, *P* < 0.001, respectively). Motor developmental delay was the most significantly relevant factor associated with catch-up head growth. The motor development index difference between the two groups was only statistically significant after adjusting for four major neonatal morbidities: IVH, BPD, sepsis and NEC status (adjusted OR 10.727, 95% CI 1.922–59.868) (Table 4).

**Table 4:** Comparison of developmental outcomes between CU and NCU groups of VLBW infants at 18–24 months of CA

	CU (*N* = 206)	NCU (*N* = 47)	*P* value	OR (95% CI)	*P* value	aOR^†^ (95% CI)	*P* value
LDI, M ± SD (points)	96.9 ± 15.3	92.7 ± 14.5	0.090				
Developmental delay, n (%)	6 (2.9)	4 (8.5)	0.076	3.101 (0.839–11.462)	0.986	1.364 (0.300–6.195)	0.688
CDI, M ± SD (points),	100.5 ± 14.3	91.8 ± 14.8	< 0.001*				
Developmental delay, n (%)	6 (2.9)	6 (12.8)	0.004*	4.878 (1.498–15.882)	0.748	2.294 (0.629–8.373)	0.209
MDI, M ± SD (points),	99.1 ± 12.8	89.7 ± 17.5	< 0.001*				
Developmental delay, n (%)	2 (1.0)	7 (14.9)	< 0.001*	17.850 (3.577–89.086)	0.013*	10.727 (1.922–59.868)	0.007*

By independent t-test, chi square test

By multiple logistic regression analysis

*Abbreviations*: *CU* Catch-up group, *NCU* No catch-up group, *OR* Odds ratio, *VLBW* Very low birth weight, *CA* Corrected age, *LDI* Language developmental index, *M* ± *SD* Mean ± Standard deviations, *CDI* Cognitive developmental index, *MDI* Motor developmental index

^*^P < 0.05 compared with CU

^†^Adjusted for IVH, BPD, Sepsis and NEC status

## Discussion

This study analyzed 253 VLBW infants with birth weight under 1,500 g at a gestation age of 23 weeks to 31 weeks. The results demonstrated that the degree of head circumference, especially that during 18**–**24 months of CA, was associated with developmental outcomes. Only a small percentage (18.6%, 47/253) of infants exhibited no catch-up head growth at 18**–**24 months of CA. The developmental outcome depended on the catch-up status at 18**–**24 months of CA.

Head circumference is a valid indicator of total brain volume and it can be used as a proxy for brain growth. Measurement of postnatal head growth as determined by the change in head circumference has been associated with total brain tissue volume and neurodevelopmental outcomes including cognition. It has been reported that severe postnatal growth failure among VLBW infants is markedly influenced by intra uterine growth and major morbidities [2–5].

In this study, the key perinatal factor determining catch-up status was the BPD. The pathophysiology that leads to infants with BPD having greater developmental delay is probably multifactorial, including chronic intermittent hypoxia, growth deficiencies, and altered environmental stimulation. First, central nervous system pathology in infants with BPD shows brain atrophy and gliosis compatible with chronic hypoxia. Second, recurrent oxygen desaturations in infants with BPD have been associated with poor weight gain which may give credence to the possibility of poor central nervous system growth. Third, environmental factors such as those associated with rehospitalization during the first year of life and feeding problems might ultimately affect mental development [16–20].

In this study, the NCU group exhibited lower developmental indices and a higher rate of developmental delay than the CU group at 18**–**24 month of CA, especially in the motor developmental index. Several studies have documented significant deleterious effects for VLBW infants with head growth failure on motor outcome, showing early impairments specifically involving eye-hand coordination and postural balance [21, 22]. The impaired control of sensory motor skills might be linked to damage in both the corticospinal tract and visual pathways. Although it is certain that head growth has an impact on the developmental outcome of these infants, multiple factors may be involved that cannot be easily quantified. Whether neonatal morbidities and the extra uterine environment with adequate nutritional support have direct or indirect effects on head growth including brain development remains unclear.

Jeng et al. (2008) have reported that the severity of BPD has a significantly negative linear relationship with motor developmental outcome in infancy after controlling for other risk factors [23]. Keunen et al. (2015) have recently analyzed the complex relationship between nutrition, neonatal morbidities, inflammation, and brain development in VLBW infants and concluded that adequate nutrition is crucial for brain growth and that nutritional therapies and supplements might benefit the developing brain [24].

VLBW infants might have less prominent, more diffuse cerebral white matter injuries that are undetectable by ultrasound but which can cause developmental disorders. The diffusion-weighted magnetic resonance imaging technique provides exquisite soft tissue differentiation. Therefore, a further study is needed to investigate the association of head circumference at 18**–**24 months of CA with developmental outcome using brain magnetic resonance imaging.

The strength of this study was that it included a prospective nationwide population-based cohort of VLBWI infants and used a newly revised developmental scale, BSID-III. This is a valuable aspect for analyzing various factors sequentially, including perinatal and postnatal factors. The results showed that developmental outcomes were significantly different between the CU and NCU groups.

This study has some limitations. Developmental outcomes result from various factors combined, making it difficult to affirm that catch-up head growth is the sole decisive cause of developmental delay. Additionally, although the standard guidelines for measuring head circumference were followed, and steps were taken to minimize intra-observer and inter-observer errors by having trained nurses perform repeated measurements, the potential for human error in the measurement process cannot be entirely ruled out, as head circumference measurements can be subjective and may vary if not consistently conducted by the same person.

In conclusion, the developmental level of a VLBW infant at 18**–**24 months of CA depended on whether head growth was caught up. Key clinical factors affecting the catch-up head growth were BPD, NEC status, length of parenteral nutrition, and ventilator care. These results showed the importance of head circumference measurement at 18**–**24 months of CA. Since infant developmental outcomes can predict school-age academic functioning, our results suggest that close follow-up and early intensive interventions are needed for VLBW infants with catch-up growth failure. Further research is needed to establish causality and explore additional factors that may influence developmental outcomes in this population.

## Data Availability

Data availability was subject to the Act on Bioethics and Safety [Law No. 1518, article 18 (Provision of Personal Information)]. The data that support the findings of this study are available from the data committee of the Korean Neonatal Network (http://knn.or.kr) and after permission from the CDC of Korea, However, restrictions apply to the availability of these data, which were used under license for the current study, and so are not publicly available. Data are available from the authors Jang Hoon Lee (neopedlee@gmail.com) upon reasonable request.

## Abbreviations

BPD: Bronchopulmonary dysplasia
BSID: Bayley scales of infant and toddler development
BW: Birth weight
CA: Corrected age
CI: Confidence interval
CU: Catch-up
DM: Diabetes mellitus
GA: Gestational age
GDM: Gestational diabetes mellitus
HTN: Hypertension
IVH: Intraventricular hemorrhage
KNN: Korean Neonatal Network
NCU: No catch-up
NEC: Necrotizing enterocolitis
NICU: Neonatal intensive care unit
PDA: Patent ductus arteriosus
PIH: Pregnancy-induced hypertension
PROM: Premature rupture of membrane
PVL: Periventricular leukomalacia
RDS: Respiratory distress syndrome
ROP: Retinopathy of prematurity
SGA: Small for gestational age
VLBW: Very low birth weight
